# Bimodal distribution and set point HBV DNA viral loads in chronic infection: retrospective analysis of cohorts from the UK and South Africa

**DOI:** 10.12688/wellcomeopenres.15941.2

**Published:** 2020-10-14

**Authors:** Louise O. Downs, Sabeehah Vawda, Phillip Armand Bester, Katrina A. Lythgoe, Tingyan Wang, Anna L. McNaughton, David A. Smith, Tongai Maponga, Oliver Freeman, Kinga A. Várnai, Jim Davies, Kerrie Woods, Christophe Fraser, Eleanor Barnes, Dominique Goedhals, Philippa C. Matthews

**Affiliations:** 1 Department of Infectious Diseases and Microbiology, Oxford Radcliffe Hospital NHS Trust, Oxford, UK; 2 Nuffield Department of Medicine, University of Oxford, Oxford, UK; 3 Division of Virology, University of the Free State, Bloemfontein, South Africa; 4 Big Data Institute, Li Ka Shing Centre for Health Information and Discovery, Oxford, UK; 5 Department of Zoology, University Of Oxford, Oxford, UK; 6 National Institute of Health Research Health Informatics Collaborative, NIHR Oxford Biomedical Research Centre, Oxford, UK; 7 Oxford University Hospitals NHS Foundation Trust, Oxford, UK; 8 Department of Virology, University of Stellenbosch, Cape Town, South Africa; 9 Nuffield Department of Population Health, University of Oxford, Oxford, UK; 10 Department of Computer Science, University of Oxford, Oxford, UK; 11 Department of Hepatology, Oxford Radcliffe Hospital NHS Trust, Oxford, UK

**Keywords:** HBV, HIV, viral load, set point, distribution

## Abstract

Hepatitis B virus (HBV) viral load (VL) is used as a biomarker to assess risk of disease progression, and to determine eligibility for treatment. While there is a well recognised association between VL and the expression of the viral e-antigen protein, the distributions of VL at a population level are not well described. We here present cross-sectional, observational HBV VL data from two large population cohorts in the UK and in South Africa, demonstrating a consistent bimodal distribution. The right skewed distribution and low median viral loads are different from the left-skew and higher viraemia in seen in HIV and hepatitis C virus (HCV) cohorts in the same settings. Using longitudinal data, we present evidence for a stable ‘set-point’ VL in peripheral blood during chronic HBV infection. These results are important to underpin improved understanding of HBV biology, to inform approaches to viral sequencing, and to plan public health interventions.

## Introduction

Hepatitis B virus (HBV) DNA viral loads (VL) show wide variation between individuals with chronic hepatitis B (CHB) infection, and are used to determine treatment eligibility ^1^. The relationship between HBV e-antigen (HBeAg)-positive status and high VL in CHB is well recognised, but there are few refined descriptions of VL distribution, and limited understanding of the biology that underpins these patterns. Set point viral load (SPVL), defined as a stable level of viraemia in peripheral blood during the initial years of chronic infection, is a concept well established in HIV ^2^. However, despite many biological similarities between HIV and HBV viral replication cycles, SPVL has not been explored for CHB to date.

Developing improved insights into the distribution of VL at a population level is important for planning wider treatment deployment to support progress towards international sustainable development goals for HBV elimination, which set ambitious targets for reducing morbidity and incidence of new CHB cases ^3^. Characterisation of HBV VL dynamics is also important for mathematical modelling, and for generating new insights into persistence, transmission and pathogenesis. To support development of *in vitro* research, understanding the VL distribution at a population level informs approaches to viral sequencing, which typically have thresholds of 10 ^3^–10 ^4^ iu/ml, below which sequences cannot be derived.

We have therefore set out to generate a preliminary description of the HBV VL distribution in independent cohorts from the UK and South Africa, to compare these patterns with VL distributions in two other chronic blood-borne viral infections, HIV-1 and hepatitis C virus (HCV), and to seek evidence for SPVL in HBV infection.

## Methods

We retrospectively collected VL measurements ± supporting metadata for adults with chronic HBV, HCV and HIV infection from four cohorts:

**(i) HBV: UK dataset**
  We collected data for adults (>18 years) with CHB infection (defined as positive HBsAg on ≥2 occasions ≥ 6 months apart) from electronic records at Oxford University Hospitals NHS Foundation Trust, as part of the National Institute of Health Research Health Informatics Collaborative (NIHR-HIC), as previously described ^4^. We assimilated VL results (Abbott M2000 platform) for 371 individuals off nucleoside analogue therapy over six years commencing 1st January 2011, for whom baseline HBeAg status was available in 351 (95%) cases. Age, sex and self-reported ethnicity (using standard ethnicity codes) were available for 352, 355 and 322 individuals, respectively. For longitudinal VL analysis, we only used data prior to commencing antiviral treatment, including patients with ≥2 measurements ≥6 months apart (n=299 individuals, 1483 timepoints). The upper limit of quantification is HBV DNA 10 ^8^ IU/ml.
**(ii) HBV: South Africa dataset**
  We collected all HBV VL data from the South African National Health Laboratory Service (NHLS) recorded over a four year period commencing 1 ^st^ January 2015 (n=6506 individuals). These were generated using various commercial platforms in different NHLS labs across the country. Other metadata (HBeAg status, HIV status, treatment data) were not available. For the purposes of analysis, we excluded VL measurements below the limit of detection based on the assumption that the majority of these samples were taken on antiviral treatment (indicated for HBV infection ± HIV co-infection). All those above the laboratory limit of quantification were designated 1.7×10 ^8^ IU/ml. For analysis of longitudinal data, we included patients with ≥2 detectable VL measurements (n=874 individuals; 9578 timepoints).
**(iii) HCV**
  Baseline HCV viral loads were collected for adults prior to commencing antiviral treatment between 2006–2018, representing 925 individuals, from the same source as the UK HBV data using the Abbott M2000 platform, and collected through the NIHR-HIC pipeline. The setting and characteristics of this study population has been previously described ^5^.
**(iv) HIV**
  HIV data were obtained from a UK database of HIV seroconverters between 1985-2014 through the BEEHIVE collaboration (n=1581) ^2^. HIV VL was measured using COBAS AmpliPrep/COBAS TaqMan HIV-1 Test, v2.0 on samples collected starting at 6-24 months after infection. SPVL was defined as the average VL for each patient over time, as previously described ^2^.

### Statistical analysis

We used Graphpad Prism v.8.2.1 for analysis of VL distributions, skewness, and univariate analysis of patient parameters associated with HBV VL (Mann Whitney U test and Kruskall Wallis test). HBV and HCV VL are conventionally reported in IU/ml, but to make direct comparisons between VL in different infections, we also converted data into copies/ml (1 IU = 5.4 copies/ml for HBV ^6^ and 2.7 copies/ml for HCV ^7^.

We used R package (version 3.6.1) to assess within and between patient VL variability, using longitudinal data from UK HBeAg-negative adults, and from South African individuals with detectable VL. A large contribution of between-host variation would provide support for SPVL. We defined total variation, between-individual and within-individual variation according to analysis of variance (ANOVA). Specifically, the calculations are as follows:

$$Variation_{Total}=\sum_{i=1}^{n}\sum_{t=1}^{n_{i}}(x_{it}-\bar{x}{)}^{2}$$

$$Variation_{Between-individual}=\sum_{i=1}^{n}n_{i}({\bar{x}}_{i}-\bar{x}{)}^{2}$$

$$Variation_{within-individual}\sum_{i=1}^{n}\sum_{t=1}^{n_{i}}(x_{it}-\bar{x}{)}^{2}$$

*n* denotes the number of individuals; *n _i_* represents the number of data points for individual *i*; *x _it_* denotes the viral load of patient *i* at time point *t*; $\bar{x}$ is the mean of viral loads of all data points; ${\bar{x}}_{i}$ is the mean of viral loads of patient *i*.

### Ethics

Data collection for the UK cohort was approved as part of the NHS Health Informatics Collaborative (NHIC Hepatitis Theme Database) by the NRES Committee South Central-Oxford C (ref: 15/SC/0523), allowing routine clinical data to be collated and analysed in anonymised form as described previously ^4, 8^. South African data collection was approved by the Health Sciences Research Ethics Committee at the University of the Free State (ref: UFS-HSD2019/0044/2603). In both cases, approval was given without the need for individual patient consent, as data were collected in anonymised form without identifying details.

## Results

**Figure 1.**
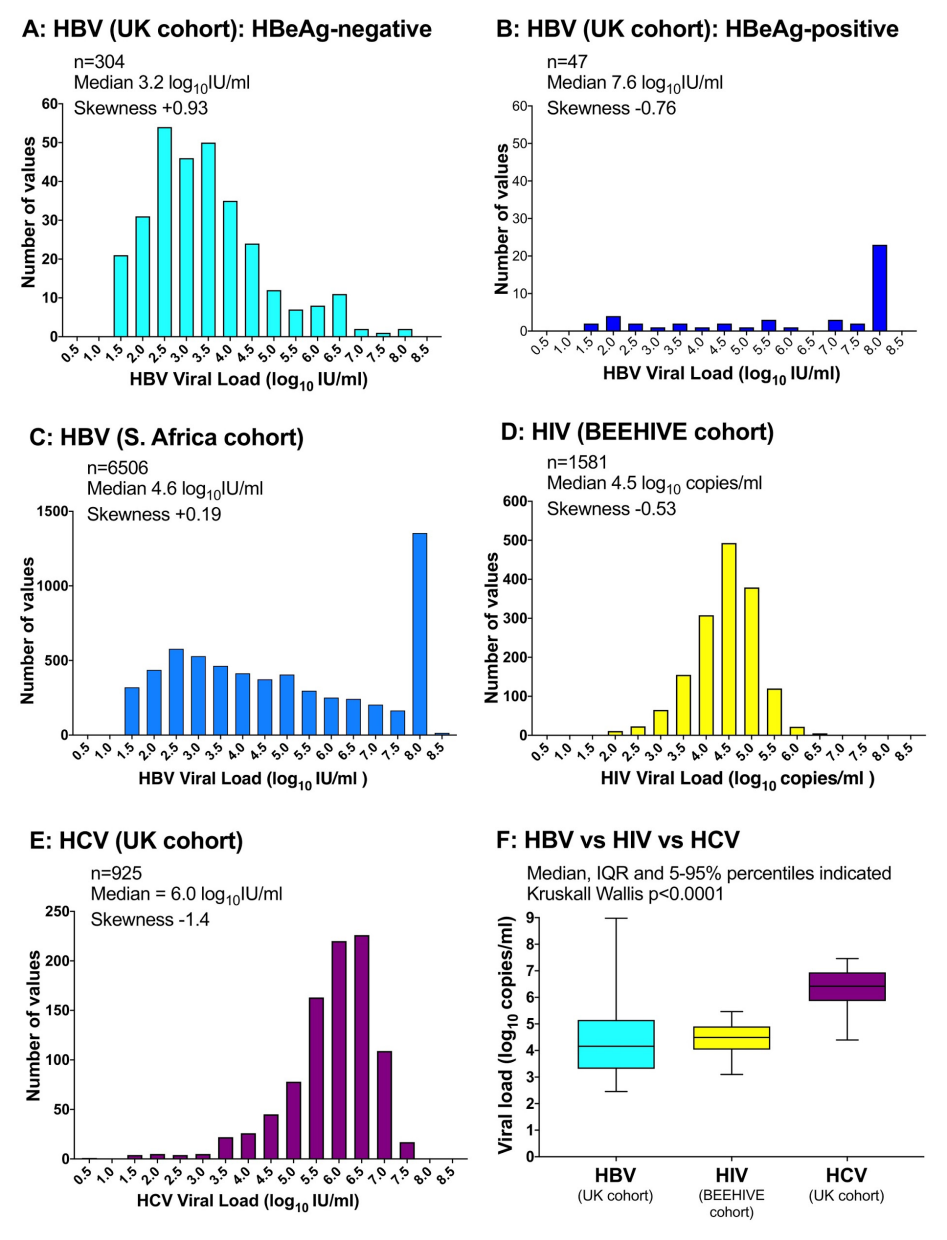
Distribution of viral loads (VL) for adults with chronic infection with Hepatitis B virus (HBV), Human Immunodeficiency Virus (HIV) and Hepatitis C virus (HCV). Panels **A**- **C** show VL distribution in HBV infection; **D** shows VL distribution in HIV infection; **E** shows VL distribution in HCV infection. Number of individuals represented, median viral load, and skewness of distribution are reported on individual panels **A**– **E**. IU/ml is standard approach to quantification for HBV and HCV (panels **A**, **B**, **C**, **E**), versus copies/ml routinely reported for HIV (panel **D**).

Our UK HBV cohort was 56% male, median age 42 years, with diverse ethnic backgrounds (among 322 individuals with self-reported ethnicity data, 38% were Asian, 34% White, 24% Black, 4% Arabic, <1% other). Overall, median HBV VL was 3.4 log _10_ IU/mL; 95% CI 3.2 – 3.5 log _10_ IU/mL (equivalent to median 4.2 log _10_ copies/ml). There was a bimodal VL distribution with two peaks:

**(i) HBeAg-negative infection** (accounting for 304/351 (87%) of measurements): median VL 3.2 log10 IU/mL (95% CI 3.0 – 3.4); right-skewed distribution ( Figure 1A; ^9^);

**(ii) HBeAg-positive infection** (accounting for 47/351 (13%) of measurements): median VL 7.6 log _10_ IU/mL (95% CI 5.6 – 8.2); left-skewed distribution ( Figure 1B; ^9^).

In the South African dataset (HBeAg status not determined), median HBV VL was 4.6 log _10_ IU/mL (95% CI 3.9 – 4.0), with a bimodal distribution and right-skew ( Figure 1C; ^9^). Median HIV VL was 4.5 log _10_ copies/mL and median HCV VL was 6.0 log _10 _IU/ml (6.4 log _10 _copies/ml), with a left skew and no bimodal distribution ( Figure 1D,E; ^9^).

For the UK data we investigated whether sex, age or ethnicity had any influence on VL; the only significant association was lower VL with increasing age in the HBeAg-positive group (p=0.01 by Kruskal Wallis, Supplementary Figure 1; extended data ^9^).

Inter-patient variation accounted for 82.7% and 88.0% of the variability in UK and South African longitudinal datasets respectively, whilst within-patient variation accounted for 17.3% and 12.0%. This provides support for a stable SPVL within individuals with CHB.

## Discussion

### Summary of Results

In this short report, we describe a consistent bimodal distribution of VL in CHB in a diverse UK population and a large South African dataset, in keeping with previously published studies (e.g. 10), and reflecting the role of HBeAg in immunomodulation ^11^. However, descriptions of this pattern have not previously been carefully refined. This is the first study to demonstrate the concept of SPVL in HBV infection, with between-host factors explaining >80% of the variation in VL during HBeAg-negative CHB.

### Inferences based on the distribution of viral loads

HBV viral loads in HBeAg-negative infection are significantly lower than HCV and HIV, which may relate to differences in viral population structure, viral fitness, host immune responses, and the availability of target cells. These factors might also explain why HIV, HCV and HBeAg-positive infection have left skew VL distributions, whereas HBeAg-negative infection has a right skew. Broadly, the biological significance of the relationship between VL and HBeAg status could be considered in two ways, first by addressing the mechanisms that underpin viraemic control, and second by considering the impact of alterations in VL on disease outcomes, including inflammatory liver disease, cancer and cirrhosis. These could not be addressed within this current dataset, but remain important questions for future research.

### Limitations and caveats

The cohorts on which we report are different in many ways (host and viral genetics, demographics, environmental factors, access to treatment and laboratory monitoring), and for this reason we do not set out to make any statistical comparisons between cohorts in different settings. Rather, we make the more general observation that in spite of these many potential differences, the overall bimodal distribution of HBV viral loads is broadly consistent. A smaller proportion of individuals with high viraemia in the UK cohort is likely to be reflective of wider access to suppressive antiviral therapy. Missing metadata is a limitation for further analysis of our South African dataset, and longer term aspirations will be to investigate larger VL datasets together with more robust longitudinal clinical and laboratory data.

### Implications for HBV sequencing

Whole genome sequencing has the potential to increase our understanding of HBV, but approximately 50% of cases fall below the current sequencing threshold ^12^. This means that at present there is a significant ‘blind spot’ in sequence data, preventing analysis of sequence variants in individuals with VL below the population median. The data presented in this report highlight the current challenges for HBV sequencing, and a need for resource investment to improve the sensitivity of sequencing approaches, for example considering amplification or enrichment approaches.

## Conclusions and future aspirations

 Enhanced descriptions of HBV VL may shed light on the biology of chronic HBV infection, inform mathematical models of viral population dynamics within and between hosts, improve understanding of risk factors for transmission and disease progression, underpin optimisation of viral sequencing methods, and help to stratify patients for clinical trials and treatment.

## Data availability

### Underlying data

Figshare: Supporting data for an analysis of HBV viral load distribution and set point in chronic infection: retrospective analysis of cohorts from the UK and South Africa. https://doi.org/10.6084/m9.figshare.11365082.v2 ^9^

This project contains the following underlying data:

- 191217 HBV VL data South Africa.xlsx (Including baseline VL data for each patient in the South African patient cohort and longitudinal values where measured)
- 200115 HBV VL metadata file.xlsx (Including baseline VL data for each patient in the UK patient cohort and longitudinal values where measured)

### Extended data

Figshare: Supporting data for an analysis of HBV viral load distribution and set point in chronic infection: retrospective analysis of cohorts from the UK and South Africa. https://doi.org/10.6084/m9.figshare.11365082.v2 ^9^

This project contains the following extended data:

- 200115 Suppl Fig 1.pdf

Supplementary Figure 1: Relationship between hepatitis B viral load, HBeAg status and (A) sex, (B) age, and (C) ethnicity, in a cohort of adults with chronic hepatitis B virus infection recruited in Oxford, UK.

Data are available under the terms of the Creative Commons Attribution 4.0 International license (CC-BY 4.0).
