# Supplementary material for: Bimodal distribution and set point HBV DNA viral loads in chronic infection: retrospective analysis of cohorts from the UK and South Africa
Source: Wellcome Open Res. 2020 Oct 14;5:113. Originally published 2020 Jun 1. [Version 2] doi: 10.12688/wellcomeopenres.15941.2 (PMC7682492; doi:10.12688/wellcomeopenres.15941.2)
Supplement: Peer review history

# Peer review: Reviewer response for version 2

**DOI:** 10.21956/wellcomeopenres.17928.r41278

**Published:** 2020-11-20

**Authors:** Gilles Wandeler

**Affiliations:** 1 Department of Infectious Diseases, Bern University Hospital, Bern, Switzerland

This is an interesting study describing HBV VL distribution in two cohorts of HBV-infected individuals, with the aim to assess inter-person and intra-person variability.

 My main remarks are the following:

- The most interesting and novel part of the paper is the analysis of variability of VL (last paragraph of the results). The authors could have gone a bit further in this analysis and its discussion. How did the inter-person and intra-person variability compare with HIV and HCV? What is the threshold of inter-personal variability needed to be able to conclude that there is a reliable set-point? How was this defined in HIV? I miss a discussion of the parallel that can be made between HIV infection (in which there is a large body research on VL set-point) and HBV. This should be expanded given that it’s the main message of the paper.
- According to the authors, a key aim of the paper is to inform sequencing approaches. But in the current version of the paper I don’t know how the results shown are helpful to this end. It is known from many studies that a large proportion of HBeAg negatives have a low VL. However, it could be interesting to discuss how the low intra-person variability could be helpful in determining TX eligibility from one single measurement. This could be important for low-income countries. Could there be any risk factors for having a very unstable VL over time? This could help determine who needs repeat VL.
- I didn’t see a justification for including the RSA cohort, as HBeAg status and other demographic/clinical characteristics are not know in this cohort. If there’s a clear reason or advantage of including data from RSA (power size for variability analysis?), then the authors should underline this. Inter and intra-person variability are evaluated based on HBeAg-neg from UK and both HBeAg-pos and neg from RSA: this “dilution” of the HBeAg aspect may bias the results. This should be discussed in the limitations.

Minor comments:

- Methods: second paragraph: it should be specified that this is about HBV VL.
- Results: Not clear from first para of text and figure if only one value per person was included or the full dataset for A and B.
- Results: Figure 1F is never mentioned in the text of the results section.
- Discussion: First para: last sentence should not be about HBeAg-neg as in RSA both HBeAg-neg and pos. are included.

Is the work clearly and accurately presented and does it cite the current literature?

Partly

If applicable, is the statistical analysis and its interpretation appropriate?

Yes

Are all the source data underlying the results available to ensure full reproducibility?

Yes

Is the study design appropriate and is the work technically sound?

Yes

Are the conclusions drawn adequately supported by the results?

Partly

Are sufficient details of methods and analysis provided to allow replication by others?

Yes

Reviewer Expertise:

HIV and viral hepatitis.

I confirm that I have read this submission and believe that I have an appropriate level of expertise to confirm that it is of an acceptable scientific standard.

# Peer review: Reviewer response for version 2

**DOI:** 10.21956/wellcomeopenres.17928.r40901

**Published:** 2020-11-02

**Authors:** Xiangjun Zhai

**Affiliations:** 1 Jiangsu Province Center for Disease Prevention and Control, Jiangsu, China

I think the revisions are appropriate.

Is the work clearly and accurately presented and does it cite the current literature?

Yes

If applicable, is the statistical analysis and its interpretation appropriate?

Partly

Are all the source data underlying the results available to ensure full reproducibility?

Yes

Is the study design appropriate and is the work technically sound?

Partly

Are the conclusions drawn adequately supported by the results?

Partly

Are sufficient details of methods and analysis provided to allow replication by others?

Yes

Reviewer Expertise:

Prevention and control of infectious diseases such as Hepatitis B, HIV, TB

I confirm that I have read this submission and believe that I have an appropriate level of expertise to confirm that it is of an acceptable scientific standard.

# Peer review: Reviewer response for version 1

**DOI:** 10.21956/wellcomeopenres.17485.r40064

**Published:** 2020-09-07

**Authors:** Xiangjun Zhai

**Affiliations:** 1 Jiangsu Province Center for Disease Prevention and Control, Jiangsu, China

1. As for the study design, the study selected 4 cohorts to study the VL distribution characteristics, but the basic characteristics of the 4 study cohorts are unclear or very different, the results are not comparable. For example, the metadata (HBeAg status, age, treatment data) were not available, but all of these characteristics are very important factors affecting the distribution of VL.
2. The definition or biological significance of the set-point viral load in chronic HBV infection patients is unclear. For example, in the tolerant phase of HBV infection, the viral load can be maintained at a high level, while in the inactive phase of HBeAg negative, the viral load can be maintained at a low level. Can the authors explain the biological significance of the set point viral load of the patients?
3. As for the Figure 1F, I don't think VL of different viruses (HBV, HCV, HIV) can be compared among the infected patients. 
4. In the discussion, the authors stated that the between-host factors explain >80% of the variation in VL during HBeAg-negative CHB. But the VL variability within and between patient was not given in the parts of results.

Is the work clearly and accurately presented and does it cite the current literature?

Yes

If applicable, is the statistical analysis and its interpretation appropriate?

Partly

Are all the source data underlying the results available to ensure full reproducibility?

Yes

Is the study design appropriate and is the work technically sound?

Partly

Are the conclusions drawn adequately supported by the results?

Partly

Are sufficient details of methods and analysis provided to allow replication by others?

Yes

Reviewer Expertise:

Prevention and control of infectious diseases such as Hepatitis B, HIV, TB

I confirm that I have read this submission and believe that I have an appropriate level of expertise to state that I do not consider it to be of an acceptable scientific standard, for reasons outlined above.

# Response

**Published:** 2020-09-11

**Authors:** Louise Downs

**Affiliations:** Oxford Radcliffe Hospital NHS Trust, UK

As for the study design, the study selected 4 cohorts to study the VL distribution characteristics, but the basic characteristics of the 4 study cohorts are unclear or very different, the results are not comparable. For example, the metadata (HBeAg status, age, treatment data) were not available, but all of these characteristics are very important factors affecting the distribution of VL.

**We agree with the reviewer about the differences between cohorts, and that we cannot determine host or viral factors that are associated with the viral load distributions we observe. We recognise that the text of our manuscript can be improved accordingly, and have made the following modifications:**

1. **We have removed the reference to ‘precise determinants’ of viral load from the abstract, and have taken out an aspiration to link VL to host characteristics from the methods, which were potentially misleading. Instead, we have simplified the abstract to say: 'While there is a well recognised association between VL and the expression of the viral e-antigen protein, the distributions of VL at a population level are not well described’. We have also improved clarity in the abstract by stating explicitly that the approach is an observational one.**
2. **A key learning point from these data is to inform HBV sequencing, which was not well represented in the introduction (although featured in the discussion); we have added this to make it clear that our intention is to provide an observation and description of HBV viraemia, rather than to make mechanistic insights or to draw direct comparisons between populations. We have amended the final sentence of the abstract to include the point about sequencing. **
3. **We have structured the discussion with sub-headings to add clarity. Reflecting the point raised by the reviewer, we have added to ‘limitations and caveats’ section to say: ‘The cohorts on which we report are different in many ways (host and viral genetics, demographics, environmental factors, access to treatment and laboratory monitoring), and for this reason we do not set out to make any statistical comparisons between cohorts in different settings. Rather, we make the more general observation that in spite of these many potential differences, the overall bimodal distribution of HBV viral loads is consistent’. **
4. **We recognise that a bigger metadata set would be of huge value, but providing this on a national level would not be feasible for any setting, and certainly not for South Africa where there are substantial clinical and laboratory resource constraints. However, our report is a very unusual opportunity in sharing viral load data for a whole country. We have added to the discussion: ‘The South African dataset represents viral load data for the whole country; assimilating wider clinical or laboratory metadata is not currently practical. In many low/middle income settings, biomarkers such as HBeAg status are infrequently measured due to resource constraints. Furthermore, linkage between clinical data (such as treatment) and laboratory data (such as viral load) is challenging at a national level for even high income settings.’ For this reason, we have formulated our observations into a short report, rather than a full length paper; we believe this is a proportionate way to share observational data which underpins questions for future research into the associations and determinants of viral load.**

The definition or biological significance of the set-point viral load in chronic HBV infection patients is unclear. For example, in the tolerant phase of HBV infection, the viral load can be maintained at a high level, while in the inactive phase of HBeAg negative, the viral load can be maintained at a low level. Can the authors explain the biological significance of the set point viral load of the patients?

**We agree this is a really interesting question. This difference in set-point according to eAg status is highlighted by Figure 1A vs 1B. The ‘biological significance’ of this observation could be considered in two ways, first addressing the mechanisms underlying the marked difference in viral loads, and second by considering the impact of this change on driving pathology. These are both complex questions, that remain to be clearly elucidated and are outside the remit of this current paper; rather than setting out to address these questions, the aim of this short report is to provide observational data that is a foundation for future research. We have added this point to the discussion.**

****

As for the Figure 1F, I don't think VL of different viruses (HBV, HCV, HIV) can be compared among the infected patients. 

**We agree that a direct comparison between viral loads is difficult, but are intended to reflect a broad comparison between the host/viral interplay for different infections.  We have amended the abstract to remove the statement that the HBV, HCV and HIV cohorts are ‘comparable’ and instead say the cohorts are ‘in the same setting’. This removes any implication that direct comparison is appropriate. We have removed panel F and the sentence that compared median viraemia that supported this figure panel in the text of the results section.**

In the discussion, the authors stated that the between-host factors explain >80% of the variation in VL during HBeAg-negative CHB. But the VL variability within and between patient was not given in the parts of results.

**The results about within/between patient variability is already included in the results, as follows: ‘Inter-patient variation accounted for 82.7% and 88.0% of the variability in UK and South African longitudinal datasets respectively, whilst within-patient variation accounted for 17.3% and 12.0%. This provides support for a stable SPVL within individuals with CHB.’ We think this provides the information that the reviewer is seeking, but would welcome further specific feedback if additional amendment is thought to be required.**


** **

- **Is the work clearly and accurately presented and does it cite the current literature?**

Yes

- **Is the study design appropriate and is the work technically sound?**

Partly

**Having improved the aims and methods to state more clearly our intention to present an observational comment about distribution of viral loads, and removing the direct comparison between HIV, HCV and HBV, we believe we have addressed any concerns.**

- **Are sufficient details of methods and analysis provided to allow replication by others?**

Yes

- **If applicable, is the statistical analysis and its interpretation appropriate?**

Partly

**As above, we think that changes to the methods and results (specific details set out above, and removal of panel 1F) tackle any deficiencies in the first version.**

- **Are all the source data underlying the results available to ensure full reproducibility?**

Yes

- **Are the conclusions drawn adequately supported by the results?**

Partly

**An improved and expanded discussion section has allowed us to present conclusions more clearly, and we have improved on objective reporting of where primary conclusions that can be drawn directly from this dataset, and where future research is still required.**
